# Supplementary material for: Nanoscale silicate melt textures determine volcanic ash surface chemistry
Source: Nat Commun. 2024 Jan 15;15:531. doi: 10.1038/s41467-024-44712-6 (PMC10789741; doi:10.1038/s41467-024-44712-6)
Supplement: Supplementary file 1 — Supplementary Information [file 41467_2024_44712_MOESM1_ESM.pdf]

Nanoscale silicate melt textures determine volcanic ash surface chemistry

Supplementary Information

Supplementary discussions 1-6

Supplementary tables 1-6

Supplementary figures 1-8

Supplementary Information references

Supplementary discussions 1-6

1) Description and preliminary interpretation of ash surface surface composition with fragmentation mode and experimental pressure and temperature
2) Discussion on the effect of boundary layer chemistry on viscosity and fracture toughness.
3) Discussion on the effect of nanophase formation in the matrix melt on viscosity and eruption triggering.
4) Natural conditions for development of Fe-Ti nanotextures
5) Comparison of results derived from different measurement techniques.
6) XPS measurement experiments and data analysis integrity

Supplementary tables 1-4

1) X-ray photoelectron spectroscopy (XPS) elemental data from the 2-10 nm surface of experimental ash particles.
2) Bulk and micro-XRF measurements of crushed and fragmented experimental materials.
3) QEMSCAN phase quantification results.
4) Mean elemental composition of identified mineral phases.
5) Comparison of EPMA measurements for matrix glass and boundary-layer glass and calculated ‘ideal’ bulk glass.
6) Calculated near-surface and bulk compositions based on combined minerology and EPMA data.

Supplementary figures 1-8

1) Variable magnification SEM-BSE images of experimental pyroclasts showing matrix textures and mineralogy in the experimental pyroclasts.
2) Ratio between average XPS surface chemistry and average bulk chemistry
3) SEM-EDX spot and linescan measurements through the compositional boundary layers surrounding pyroxene microlites
4) Comparison of textures between pyroclasts formed at room temperature and 850 ^{o}C.G
5) A comparison of clast types and textures in natural (a-c) and experimental (d-f) pyroclasts.
6) Textures of natural pyroclastic fallout samples.
7) Qualitative SEM-BSE observations of fracture pathways in experimental pyroclasts.
8) Comparison of average elemental concentration from methods used in the study.

Supplementary discussion 1.

Description and preliminary interpretation of ash surface surface composition with fragmentation mode and experimental pressure and temperature

Variations in particle surface chemistry with fragmentation mode, P and T are generated from the interaction of the physical (i.e. size, shape and distribution) and mechanical properties of constituent phases with the stress field and fracture energy released during the fragmentation process[1].

Direct comparison of the bulk and nanoscale surface measurements in Supplementary Figure 8 suggest that the depth dependence for concentration (i.e., the difference between XPS measurements and microscale or bulk measurements) and the sensitivity to fragmentation mode varies for different elements. The most depth-dependent elemental concentrations appear to be Mg, K, Na and Fe, in that order. These elements also show variations in concentration in the EDS-linescans between compositional boundary layers and microlite-bearing glass in Supplementary Figure 3. A dependence on fragmentation mode is found for all elements, but is most pronounced and consistent for Mg, Na and K where, in all cases, crushed samples have higher concentration at the nm-scale than samples fragmented by rapid decompression under the same conditions. This pattern is also found at the micron scale for K, but not Mg or Na (see Figure 2a, c and Supplementary Figure 8).

Plagioclase is a phase that is depleted at particle boundaries for all experiments, in line with similar observations in natural samples[2], but we observe higher fractions of (Na-rich) plagioclase (see Supplementary Table 3 for phase compositions) and a corresponding increase in Na at particle surfaces formed by crushing. Changes in the experimental conditions show no consistent effect with pressure, while higher temperature is associated with slight enrichment of Fe.

We note that lower fracture energies during fragmentation result in an increased sensitivity of fracture paths to mechanical properties and material texture[3], and we infer that higher fracture energy is imparted during shock tube experiments, but further focused experimental and analytical work is required to interpret and classify variations in surface composition generated from varying fragmentation processes and P-T conditions.

Supplementary discussion 2.

Effect of boundary layer chemistry on viscosity and fracture toughness.

As a first-order approximation, the boundary layer contains fewer network-modifying cations (Mg and Fe) and more Al^{3+} charge-compensating alkalis (Na and K)[4] than the matrix glass, therefore it is expected to increase polymerisation and lower the fracture toughness. Accurate chemical analysis is hampered by activation volumes (SEM-EDS) and alkali migration (EPMA), however the results obtained for our EPMA results can be used to calculate a lower estimate for the variation in NBO/T between boundary layer and matrix glasses. Using a pre-eruptive magma temperature of 1000 °C, pressure of 200 MPa and fO_2 of NNO+2, as constrained by Andujar et al.[5] and Samaniego et al.[6], we calculate an Fe^{3+}/total Fe ratio of 0.4 using the method of Kress and Carmichael[7]. Using these values, we calculate the average degree of polymerisation as the ratio of non-bridging oxygens to tetrahedrally-coordinated cations (NBO/T[8]) as 0.18 for the compositional boundary layer glass and 0.24 for the matrix glass. We also estimate the melt viscosity of the boundary layer to be at least 0.5 log Pa s more viscous than the matrix glass melt using the GRD viscosity calculator[9], however the effect of preferential fracturing through Maxwell criteria10 is inferred to be negligible since we see the same magnitude of surface chemistry variation in samples fragmented well above T_g (estimated using the GRD calculator between 669-676 °C for all measured and calculated glass compositions) and at 25 °C. These modest variations in NBO/T and viscosity may be diminished by reduction in the elastic moduli[11] for the element enrichment and depletion we observe. In summary, we consider this to be a second order effect on the observed localisation of fractures in the boundary layers compared to the orders of magnitude variations in fracture toughness that may be imparted by the nanotexture in the matrix glass[12,13].

Supplementary discussion 3.

Discussion on the effect of unmixing or nanolitization on remnant melt viscosity and eruption triggering.

In high-resolution SEM-BSE images, matrix textures appear to show that changes in melt .In particular the coarsening of the features surrounding plagioclase microlites (in agreement with prior observations[14]) and the fining and finally disappearance of the features in compositional boundary layers surrounding pyroxenes crystals (Fig. 3b-c, Supplementary Figures 2-6) provide evidence for the control of local melt chemistry on nanophase size.licate liquid unmixing, crystal-liquid and two-liquid partition coefficients favour partitioning of Fe, Mg, Ca and Ti into

the nanophase[15]. The partitioning of Fe and network modifiers, such as Mg and Ca, into a discrete nanophase will cause an increase in the viscosity of the residual interstitial melt[16,17]. A rapid increase in melt viscosity can trigger a transition from viscous flow to brittle failure[18], and thus the onset of nanolitization or silicate liquid immiscibility may be an effective and sudden eruption trigger[12,19–21.]

Supplementary discussion 4

Natural conditions for development of Fe-Ti melt nanotextures

The development of Fe(Ti) nanolites is controlled by the oxidation state and coordination of Fe (determined by the oxygen fugacity[22]) the concentration of Fe[23] in a silicate melt and the undercooling. The injection of relatively mafic, hot and volatile-rich magma into a more evolved and cooler magma body (known as mafic recharge) is established as a common triggering process for volcanic eruptions[24,25], including at Tungurahua[6,26], and a potential source for oxidizing fluids (particularly water) that increase oxygen fugacity and generate high ΔT that may trigger nanolite crystallization[27] and silicate liquid immiscibility in andesitic magmas[28,29]. Oxygen fugacity conditions increasing from QFM+1 to QFM+2 are shown to favour nanolite crystallization[27]; a range of QFM+1.5-3 in a high-Fe andesite is modelled to promote silicate liquid immiscibility[30]. These conditions are in the range for arc tectonic settings, although not for plume or mid-ocean ridge volcanism[31]. For nanolites, timescale of nanolite formation are highly dependent on cooling rate, but vary from >1000 minutes (>17 hours) for rhyolite[32] to the first 100s of seconds of cooling for basaltic compositions at high cooling rates[20,33]. For SLI, in the case of binodal CBL-triggered unmixing, timescales may be governed by diffusion rates in the melt[14] with unmixing textures maturing over timescales likely longer than for nanolite formation.

In addition to the basaltic and rhyolitic examples in the main text, the nanotextures documented in this study have been recently recorded in andesitic (Shinmoedake[34]), trachyandesitic (Tambora[20]), and trachytic (Fukutoku-oka-no-Ba[27]) volcanic eruption products.

Supplementary discussion 5.

Comparison of results derived from different measurement techniques.

We use four analytical techniques in the paper: micro-XRF, SEM-EDS and BSE (QEMSCAN), EPMA and XPS to produce three complementary datasets involving different resolution: micro-XRF (bulk chemistry), QEMSCAN+EPMA (microscale surface and bulk chemistry), and XPS (nanoscale surface chemistry). The average results for each sample and each technique (bulk QEMSCAN+EPMA chemistry) are shown in Supplementary Figure 8.

We note some observations from these comparative results.

1. QEMSCAN+EPMA and micro-XRF measurements are in good agreement for all elements

2. Maximum differences between XPS and the other measurements modes for Ca and Al are less than 20%, whereas for Fe and Na differences are up to ~180% and for Mg reach ~800%.

3. The zig-zag patterns of relatively increasing or decreasing concentration between samples shown below are reproduced in both XPS and QEMSCAN+EPMA data for Al, Ca, Fe, and K, but not for Mg and Na. For these latter two elements, the XPS data shows the same relative concentration patterns as for K, but the EPMA+QEMSCAN data are relatively flat.

The similarity of the results in terms of absolute concentration (Al and Ca) and relative concentration between crushed and shock tube samples (Al, Ca and K) in Supplementary Figure 8 shows that a systematic bias is not present between the different measurement techniques. To our knowledge, biases affecting Mg, Fe, and Na (points 2 and 3) in SEM-EDS+EPMA are the well-known glass measurement issues of Na-mobility (and time-dependent depletion in measurements) and Fe oxidation state uncertainty, however we estimated glass chemistry by difference instead of relying on EPMA measurements (see Methods).

EPMA chemistry determination and SEM-based phase mapping are discipline standards in Earth science and the mining industry, respectively, and synthesis of these data types is common in the literature. XPS is a newer technique, but has been increasingly employed in the Earth sciences and other fields in the past decades, and comparisons between nanoscale surfaces measured by XPS and bulk compositions measured by other techniques have been made in numerous studies on volcanic products[35–45.]

Supplementary discussion 6.

XPS measurement experiments and data analysis integrity

XPS quantification is based on the measurement of peak areas above the spectrum background. The intensity or area (A) of the peaks, depends on the photon flux (J), the concentration of the atom/ion in the solid (ρ), the cross-section (σ) for photoelectron emission (which depends on the element and energy being considered), instrumental factors (K), and the electron attenuation length (λ), (A= J.ρ.σ.K.λ). In practice, atomic sensitivity factors (F) containing J, σ K λ, are used, and the quantification is given as the fraction of each peak area divided by the sensitivity factor, normalised for all peaks. [i] atomic % = {(Ai/Fi)/Σ(A/F)} where Ai and Fi are the peak area and sensitivity factor of element i. and Σ(A/F) is the sum of the peak area/sensitivity factor ratios for all elements. Therefore, the measurement uncertainty basically springs from how well the peak area is measured, i.e., how well the peak is acquired (high signal to noise ratio is needed) and how well the background is subtracted. For data analysis we used the software casaXPS (SINTEF's licence) and for the quantification we used the whole spectrum area i.e. no peak deconvolution was performed. Therefore, quantification, was not dependent on peak fitting something that contains a significant degree of uncertainty.

To make sure that no errors were introduced due to instrumental factors all samples were measured on the same instrument and the same vacuum level. The spectra were acquired at the same angle of emission (0°, vertical emission) and the same analyser acceptance angle. In all samples, each peak corresponding to a specific element was acquired at the same acquisition time and the same background type was subtracted for the same peak in all samples. For the quantification we used the instrument provider Wagner sensitivity factors stored in the spectrometer's library. These are empirical and very reliable as are based on measurements performed on standards. The same X-ray power was used for all measurements, all high-resolution peaks were acquired at the same pass energy. For the quantification we measured areas of peaks having the same energy to eliminate discrepancies arising from attenuation length differences. As an example, we compared the Mg 2*p* peaks in all samples and not Mg 1*s* in one sample with the Mg 2*p* in another sample. The Mg 1*s* photoelectron has lower attenuation length and its signal originates from the outermost surface, whilst the Mg 2*p* has higher attenuation length and originates from slightly deeper in the sample. Thus, we compare the elemental content at the same depth for all samples. Hower, the analysis depth of XPS of <10 nm is 2-3 orders of magnitude smaller than that of XRF/EDS/EPMA and this difference in

scale makes the comparison between surface and bulk evident.

To ensure that no errors attributed to the design of the experiment were introduced in the XPS measurements, the following measures were taken. We analysed 5 different areas of each sample. These gave identical spectra ensuring measurement reproducibility. Each sample was tested for irradiation induced diffusion of alkaline elements (K, Na). For this we performed time resolved experiments by acquiring survey spectra at different acquisition times 1, 5 and 10 minutes. The results showed no such dependence as the spectra exhibited the same peak intensity ratios. To ensure absence of irradiation effects throughout the whole measurement duration for each sample, spectra acquisition started and ended with acquiring survey spectra with the same acquisition parameters. The comparison showed no irradiation effects as the peak intensity ratios of the first and last survey spectrum of the same sample were identical.

Supplementary Tables 1-6.

Supplementary Table 1. X-ray photoelectron spectroscopy (XPS) elemental data from the 2-10 nm surface of experimental ash particles. Shown for each sample with 1 standard deviation. Acquisition details provided in the Methods.

	**XPS Sample Composition (at. %)**							
**Element**	**RT10C**	**RT10F**	**RT30C**	**RT30F**	**HT10C**	**HT10F**	**HT30C**	**HT30F**
**Al**	7.79 ± 0.07	8.64 ± 0.14	7.24 ± 0.57	8.38 ± 0.32	7.12 ± 0.89	7.75 ± 0.33	7.01 ± 0.48	6.87 ± 0.18
**Ca**	2.63 ± 0.02	2.85 ± 0.08	2.41 ± 0.08	2.48 ± 0.11	2.56 ± 0.07	2.17 ± 0.07	2.24 ± 0.19	1.99 ± 0.09
**Fe**	1.05 ± 0.06	1.00 ± 0.02	1.29 ± 0.07	1.06 ± 0.13	1.65 ± 0.07	1.68 ± 0.13	1.46 ± 0.12	1.54 ± 0.12
**K**	0.96 ± 0.02	0.83 ± 0.02	0.95 ± 0.09	0.88 ± 0.09	1.03 ± 0.04	0.96 ± 0.03	1.05 ± 0.08	1.04 ± 0.06
**Mg**	0.66 ± 0.02	0.33 ± 0.08	0.94 ± 0.04	0.31 ± 0.06	0.90 ± 0.14	0.29 ± 0.09	0.83 ± 0.05	0.44 ± 0.07
**Mn**	n.d	n.d	n.d	n.d	n.d	n.d	n.d	n.d
**Na**	3.71 ± 0.17	2.48 ± 0.14	4.57 ± 0.19	2.90 ± 0.24	4.96 ± 0.62	3.49 ± 0.22	5.11 ± 0.20	3.79 ± 0.23
**O**	63.25 ± 0.47	62.28 ± 0.64	63.95 ± 0.51	62.86 ± 0.54	63.80 ± 1.71	63.07 ± 0.61	63.66 ± 0.67	64.25 ± 0.58
**P**	n.d	n.d	n.d	n.d	n.d	n.d	n.d	n.d
**Si**	19.93 ± 0.54	21.59 ± 0.55	18.63 ± 0.49	21.13 ± 0.35	17.98 ± 1.51	20.59 ± 0.65	18.63 ± 0.51	20.09 ± 0.32
**Ti**	n.d	n.d	n.d	n.d	n.d	n.d	n.d	n.d

Supplementary Table 2. Bulk and micro-XRF measurements of crushed and fragmented experimental materials. Due to small sample sizes for fragmented samples, bulk XRF was not possible. However, micro-XRF measurements were conducted for all samples using documented instrument setup and methods[53]. See Methods for details of both techniques.

Element	Micro XRF Concentration (at. %)								
	RT10C	RT30C	RT10F	RT30F	HT10C	HT30C	HT10F	HT30F	Av. ± 1 SD
**Al**	7.04	6.95	7.37	7.31	7.08	6.78	6.97	6.84	7.04 ± 0.21
**Ca**	2.73	2.70	2.95	2.94	2.71	2.59	2.63	2.58	2.73 ± 0.14
**Fe**	1.81	1.83	1.79	1.78	1.95	1.88	1.81	1.90	1.84 ± 0.06
**K**	0.73	0.76	0.65	0.66	0.72	0.79	0.77	0.80	0.74 ± 0.06
**Mg**	1.39	1.40	1.43	1.45	1.46	1.45	1.34	1.35	1.41 ± 0.05
**Mn**	n.d	n.d	n.d	n.d	n.d	n.d	n.d	n.d	
**Na**	3.20	3.21	3.21	3.21	3.22	3.19	3.24	3.21	3.21 ± 0.01
**O**	61.92	61.92	61.82	61.82	61.87	61.95	61.94	61.96	61.90 ± 0.06
**P**	n.d	n.d	n.d	n.d	n.d	n.d	n.d	n.d	
**Si**	20.94	20.99	20.57	20.62	20.75	21.11	21.05	21.10	20.89 ± 0.22
**Ti**	0.24	0.25	0.22	0.22	0.24	0.26	0.25	0.26	0.24 ± 0.02

Element	Bulk XRF concentration (at. %)								
	RT10C	RT30C	RT10F	RT30F	HT10C	HT30C	HT10F	HT30F	Av. ± 1.SD
**Al**	7.29	7.07	-	-	7.15	7.08	-	-	7.15 ± 0.10
**Ca**	2.70	2.64	-	-	2.66	2.65	-	-	2.66 ± 0.03
**Fe**	1.84	1.92	-	-	1.91	1.92	-	-	1.9 ± 0.04
**K**	0.77	0.77	-	-	0.76	0.76	-	-	0.77 ± 0.01
**Mg**	2.05	2.27	-	-	2.20	2.27	-	-	2.20 ± 0.10
**Mn**	n.d	n.d	-	-	n.d	n.d	-	-	n.d
**Na**	2.69	2.63	-	-	2.64	2.63	-	-	2.65 ± 0.03
**O**	62.09	62.27	-	-	62.06	62.21	-	-	62.16 ± 0.10
**P**	n.d	n.d	-	-	n.d	n.d	-	-	n.d
**Si**	20.62	20.52	-	-	20.60	20.52	-	-	20.57 ± 0.05
**Ti**	0.24	0.24	-	-	0.25	0.24	-	-	0.24 ± 0.00

Supplementary Table 3. QEMSCAN phase quantification results. The mean fraction for both the bulk (Total) and µm-scale surfaces (Surface) for all particles >9 µm diameter are presented. The absolute difference (Abs. Diff.) and relative difference (Rel. Diff.) between the bulk and the surface is given below the phase fractions for each sample, and the standard error of the mean ($\sigma_{\bar{x}}$) is given below each value. Acquisition details are provided in the Methods.

	Phase	Total glass	Total plagioclase	Pyroxene microlites	Ortho-pyroxene	Clino-pyroxene	Quartz	Olivine
**RT10C**	Total %	43.18 ± 0.47	38.32 ± 0.53	11.28 ± 0.19	1.33 ± 0.13	3.85 ± 0.24	0.06 ± 0.10	0.39 ± 0.06
	Surface %	40.41 ± 0.74	41.51 ± 1.14	10.26 ± 0.24	2.08 ± 0.33	5.09 ± 0.67	0.01 ± 0.01	0.42 ± 0.14
**RT10F**	Total %	38.74 ± 0.48	42.10 ± 0.52	9.00 ± 0.17	3.30 ± 0.23	3.93 ± 0.24	0.04 ± 0.04	0.32 ± 0.05
	Surface %	31.96 ± 0.76	49.68 ±1.22	7.95 ± 0.23	4.71 ± 0.64	4.56 ± 0.67	0.01 ± 0.04	0.60 ± 0.20
**RT30C**	Total %	43.24 ± 0.48	38.11 ± 0.64	11.57 ± 0.21	2.88 ± 0.19	2.34 ± 0.24	0.06 ± 0.02	0.20 ± 0.05
	Surface %	40.15 ± 0.78	41.19 ± 1.17	10.46 ± 0.27	5.21 ± 0.60	2.37 ± 0.50	0.01 ± 0.01	0.38 ± 0.17
**RT30F**	Total %	43.81 ± 0.56	36.79 ± 0.55	10.72 ± 0.24	2.74 ± 0.14	3.10 ± 0.36	0.13 ± 0.12	0.51 ± 0.07
	Surface %	33.15 ± 0.97	50.06 ± 1.51	9.01 ± 0.32	3.67 ± 0.69	2.92 ± 0.70	0.02 ± 0.01	0.68 ± 0.25
**HT10C**	Total %	46.96 ± 0.67	36.86 ± 0.70	11.54 ± 0.24	1.70 ± 0.27	1.84 ± 0.14	0.07 ± 0.01	0.18 ± 0.02
	Surface %	42.26 ± 0.95	40.63 ± 1.33	11.07 ± 0.31	2.68 ± 0.53	2.57 ± 0.52	0.02 ± 0.00	0.47 ± 0.16
**HT10F**	Total %	41.63 ± 0.44	39.47 ± 0.57	10.11 ± 0.18	2.84 ± 0.16	2.58 ± 0.17	0.06 ± 0.06	0.11 ± 0.11
	Surface %	36.01 ± 0.24	46.73 ± 0.45	8.99 ± 0.06	4.23 ± 0.16	3.09 ± 0.17	0.01 ± 0.00	0.04 ± 0.00
**HT30C**	Total %	49.45 ± 0.55	31.71 ± 0.67	10.96 ± 0.21	1.72 ± 0.16	4.05 ± 0.27	0.09 ± 0.10	0.27 ± 0.02
	Surface %	46.15 ± 0.88	35.10 ± 1.26	9.89 ± 0.24	3.29 ± 0.58	4.78 ± 0.77	0.01 ± 0.00	0.45 ± 0.18
**HT30F**	Total %	51.39 ± 0.45	31.52 ± 0.47	10.61 ± 0.18	1.31 ± 0.14	2.21 ± 0.19	0.16 ± 0.15	0.47 ± 0.02
	Surface %	47.21 ± 0.76	37.07 ± 1.03	9.71 ± 0.24	1.99 ± 0.32	2.43 ± 0.49	0.07 ± 0.02	1.05 ± 0.19

Supplementary Table 4. Mean elemental composition of identified mineral phases. Data are presented as atomic %, including 1 SD error. Oxygen is calculated via difference. The full EPMA dataset is available in Supplementary data 2 together with the measurement protocol and calibration standards.

**Element**	**EPMA Concentration (at. %)**					
	**Plagioclase (n87)**	**Pyroxene Microlites (n19)**	**Augite (n27)**	**Ortho-pyroxene (n23)**	**Olivine (n11)**	**Fe-Ti phenocrysts n(5)**
**Al**	11.36 ± 0.45	1.41 ± 0.35	1.01 ± 0.10	0.49 ± 0.17	0.06 ± 0.07	2.21 ± 0.18
**Ca**	4.15 ± 0.42	2.96 ± 0.83	7.68 ± 0.29	0.74 ± 0.14	0.13 ± 0.05	0.08 ± 0.03
**Fe**	0.21 ± 0.04	4.75 ± 0.45	3.20 ± 0.61	5.11 ± 0.50	7.28 ± 0.16	36.43 ± 0.44
**K**	0.16 ± 0.05	0.10 ± 0.10	0.01 ± 0.01	0.00 ± 0.00	0.01 ± 0.01	0.03 ± 0.02
**Mg**	0.05 ± 0.02	10.79 ± 0.74	8.42 ± 0.62	13.93 ± 0.74	20.62 ± 0.31	2.83 ± 0.68
**Mn**	0.00 ± 0.00	0.12 ± 0.02	0.08 ± 0.02	0.12 ± 0.02	0.11 ± 0.01	0.11 ± 0.02
**Na**	3.39 ± 0.37	0.26 ± 0.22	0.29 ± 0.09	0.04 ± 0.08	0.01 ± 0.02	0.07 ± 0.07
**O**	61.60	60.14	59.97	60.01	57.35	53.67
**P**	0.01 ± 0.01	0.03 ± 0.01	0.01 ± 0.01	0.00 ± 0.00	0.03 ± 0.02	0.00 ± 0.00
**Si**	19.05 ± 0.53	19.21 ± 0.55	19.16 ± 0.37	19.45 ± 0.21	14.38 ± 0.35	0.48 ± 0.62
**Ti**	0.02 ± 0.01	0.23 ± 0.04	0.19 ± 0.04	0.09 ± 0.03	0.01 ± 0.01	4.09 ± 0.15

Supplementary Table 5. Comparison of EPMA measurements for matrix glass and boundary-layer glass and calculated 'ideal' bulk glass. Due to the defocused (10 μm) beam diameter and pervasive micro- to nanoscale heterogeneities in the matrix, the EPMA measurements (Supplementary Data 3) likely include variable contributions from nanolites or immiscible globules, microlites and compositional gradients from diffusive boundary layers. Therefore, we calculate an 'ideal' bulk glass composition (i.e., equivalent to the glass composition including nanoscale phases that are below QEMSCAN resolution) that we fit to the bulk XRF data (Supplementary Table 2) using iterative goal-seeking in Microsoft Excel based on measured phase fractions (Supplementary Table 4) and crystalline phase compositions (Supplementary Table 5). The predicted bulk composition and measured bulk composition are shown in the right-hand columns. at. % is atomic %, avg. is average.

**Element at. %**	**EPMA points**		**Ideal bulk glass**	**Relative to avg. XRF**	
	**Boundary Layer Glass (n=7)**	**Matrix Glass (n=23)**		**Predicted**	**Measured**
**Al**	6.34	6.77	5.77	7.12	7.15
**Ca**	1.48	2.04	0.97	2.65	2.66
**Fe**	1.56	1.73	2.33	1.89	1.90
**K**	1.46	1.07	1.65	0.76	0.77
**Mg**	0.60	1.16	0.22	2.20	2.20
**Mn**	0.10	0.11	-	0.02	0.11
**Na**	3.07	3.04	2.99	2.63	2.65
**O**	62.56	62.29	63.15	61.80	62.16
**P**	0.16	0.12	-	0.01	0.22
**Si**	22.43	21.49	22.45	20.44	20.57
**Ti**	0.35	0.30	0.48	0.23	0.24

Supplementary Table 6. **Calculated near-surface and bulk compositions based on combined minerology and EPMA data.** Error is 1 standard error, calculated from the propagation of all errors in EPMA measurements. *indicates that oxygen is a composite derived from multiple elements. Calculations include the hypothetical glass component.

Elem-ent	Calculated near surface composition (at. %)							
	**RT10C**	**RT10F**	**RT30C**	**RT30F**	**HT10C**	**HT10F**	**HT30C**	**HT30F**
**Al**	7.05 ± 1.25	7.21 ± 1.39	7.03 ± 1.24	6.91 ± 1.17	7.05 ± 1.25	7.08 ± 1.28	6.66 ± 0.99	6.73 ± 0.99
**Ca**	2.65 ± 0.49	2.72 ± 0.52	2.55 ± 0.47	2.53 ± 0.45	2.65 ± 0.49	2.56 ± 0.47	2.45 ± 0.39	2.30 ± 0.36
**Fe**	1.85 ± 0.11	1.87 ± 0.1	1.89 ± 0.11	1.91 ± 0.11	1.85 ± 0.11	1.86 ± 0.10	1.99 ± 0.12	2.04 ± 0.11
**K**	0.78 ± 0.02	0.72 ± 0.02	0.79 ± 0.02	0.79 ± 0.02	0.78 ± 0.02	0.76 ± 0.02	0.88 ± 0.02	0.91 ± 0.02
**Mg**	1.92 ± 0.17	1.95 ± 0.15	2.01 ± 0.18	2.02 ± 0.17	1.92 ± 0.17	1.85 ± 0.15	1.95 ± 0.17	1.75 ± 0.14
**Mn**	0.02 ± <0.01	0.02 ± <0.01	0.02 ± <0.01	0.02 ± <0.01	0.02 ± <0.01	0.02 ± <0.01	0.02 ± <0.01	0.02 ± <0.01
**Na**	2.63 ± 0.37	2.62 ± 0.41	2.62 ± 0.37	2.59 ± 0.35	2.63 ± 0.37	2.62 ± 0.38	2.59 ± 0.31	2.64 ± 0.31
**O**	61.00*	60.52*	61.01*	60.61*	61.00*	60.14*	60.99*	60.77*
**P**	0.01 ± <0.01	0.01 ± <0.01	0.01 ± <0.01	0.01 ± <0.01	0.01 ± <0.01	0.01 ± <0.01	0.01 ± <0.01	0.01 ± <0.01
**Si**	20.21 ± 4.30	19.89 ± 4.56	20.23 ± 4.29	20.10 ± 4.10	20.21 ± 4.30	19.87 ± 4.31	20.40 ± 3.65	20.33 ± 3.60
**Ti**	0.25 ± <0.01	0.24 ± <0.01	0.25 ± <0.01	0.25 ± <0.01	0.25 ± <0.01	0.25 ± <0.01	0.28 ± <0.01	0.29 ± <0.01
**Elem-ent**	**Calculated bulk composition (at. %)**							
**Al**	7.24 ± 1.38	7.68 ± 1.73	7.21 ± 1.36	7.78 ± 1.77	7.24 ± 1.38	7.57 ± 1.61	6.85 ± 1.11	7.11 ± 1.21
**Ca**	2.62 ± 0.50	3.00 ± 0.65	2.83 ± 0.54	2.97 ± 0.66	2.62 ± 0.50	2.90 ± 0.61	2.49 ± 0.42	2.46 ± 0.43
**Fe**	1.88 ± 0.11	1.74 ± 0.10	1.84 ± 0.11	1.63 ± 0.09	1.88 ± 0.11	1.70 ± 0.09	2.01 ± 0.11	1.92 ± 0.10
**K**	0.74 ± 0.02	0.61 ± 0.02	0.74 ± 0.02	0.64 ± 0.02	0.74 ± 0.02	0.68 ± 0.02	0.83 ± 0.02	0.85 ± 0.02
**Mg**	2.19 ± 0.19	2.12 ± 0.16	2.09 ± 0.18	1.93 ± 0.14	2.19 ± 0.19	1.87 ± 0.14	2.22 ± 0.19	1.89 ± 0.14
**Mn**	0.02 ± <0.01	0.02 ± <0.01	0.02 ± <0.01	0.02 ± <0.01	0.02 ± <0.01	0.02 ± <0.01	0.02 ± <0.01	0.02 ± <0.01
**Na**	2.65 ± 0.41	2.68 ± 0.49	2.64 ± 0.40	2.72 ± 0.50	2.65 ± 0.41	2.70 ± 0.46	2.61 ± 0.34	2.7 ± 0.37
**O**	61.81*	61.60*	61.79*	61.54*	61.81*	61.41*	61.83*	61.75*
**P**	0.01 ± <0.01	0.01 ± <0.01	0.01 ± <0.01	0.01 ± <0.01	0.01 ± <0.01	0.01 ± <0.01	0.01 ± <0.01	0.01 ± <0.01
**Si**	20.40 ± 4.64	20.05 ± 5.37	20.39 ± 4.62	20.08 ± 5.43	20.40 ± 4.64	20.13 ± 5.10	20.58 ± 4.00	20.53 ± 4.18
**Ti**	0.24 ± <0.01	0.20 ± <0.01	0.24 ± <0.01	0.20 ± <0.01	0.24 ± <0.01	0.22 ± <0.01	0.26 ± <0.01	0.26 ± <0.01

Supplementary Figures 1-7

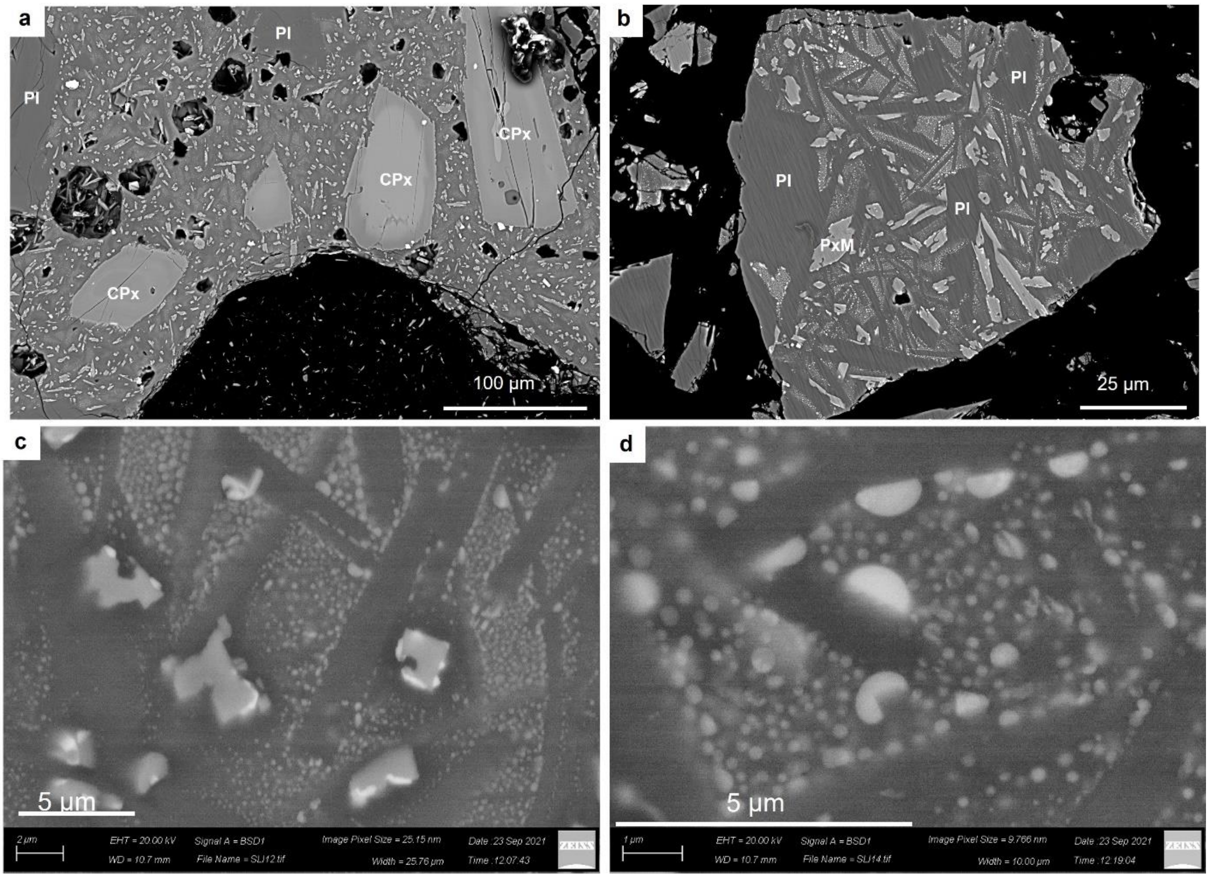

Supplementary Figure 1. **Variable magnification SEM-BSE images of experimental pyroclasts showing matrix textures and mineralogy in the experimental pyroclasts**. a) BSE image of the lava block prior to any experimental treatment. Diffuse lighter patches can be seen in the matrix glass. Plagioclase (Pl) and zoned subhedral clinopyroxene (CPx) phenocrysts can be seen in a highly crystalline matrix. Field of view (FOV) is ca. 400 × 300 µm. b) A clast from a sample crushed without experimental heating. The matrix glass can be seen to host small, rounded bright features. The clast contains abundant plagioclase microlites and pigeonite (Ca-poor clinopyroxene) microlites (PxM). FOV is ca. 125 × 100 µm. c) The matrix is comprised of a population of euhedral lathe-like plagioclase microlites and two populations of pyroxene microlites (PxMs), ~80% pigeonite and ~20% enstatite, in a glass hosting a high number density of high-BSE-intensity nanoscale features. All pyroxene microlites have a darker band of glass surrounding them that is free of thethese nanoscale features. d) A higher-magnification image showing bright hemispherical objects up to 1 µm in diameter apparently wetting the surface of plagioclase microlites, with smaller rounded and circular examples suspended in the matrix glass. These observations suggest that Fe-rich globules formed via silicate liquid immiscibility comprise an appreciable fraction of the nanoscale Fe-rich phases found in the Tungurahua ash.

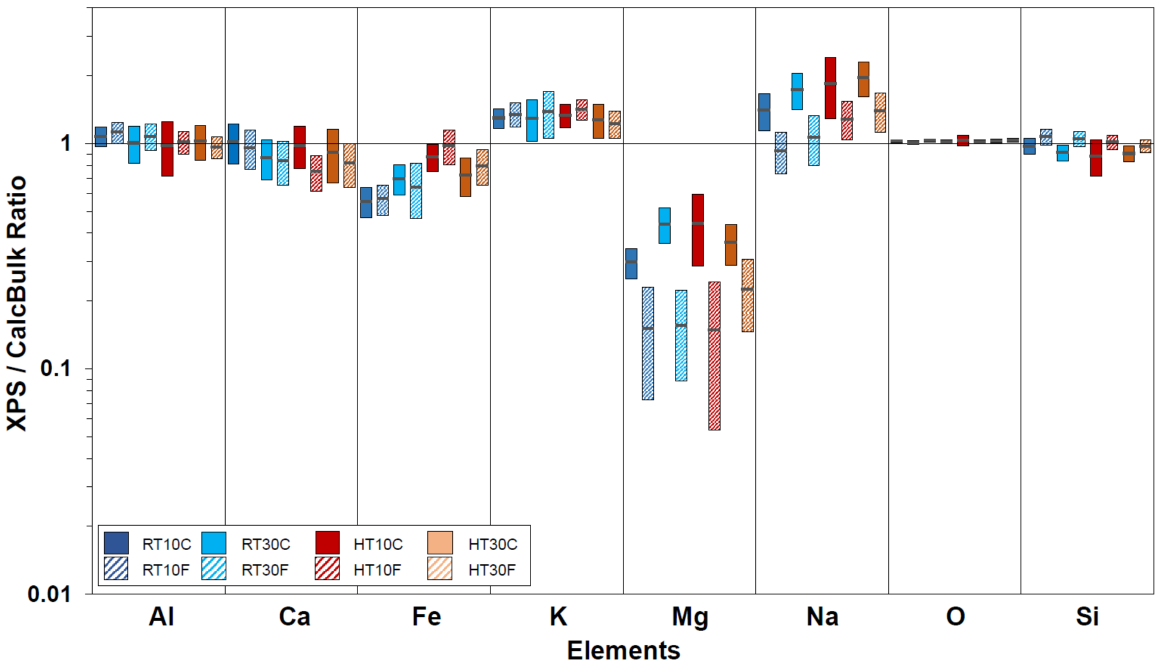

Supplementary Figure 2. **Ratio between average XPS surface chemistry and average bulk chemistry.** Bulk chemistry was calculated by combining EPMA analysis (see Supplementary Tables 3-4) and average modal phase fractions measured by QEMSCAN (see Methods for further details) are plotted for major elements. Each coloured bar shows data from one experiment, where bar height = 2 × standard error.

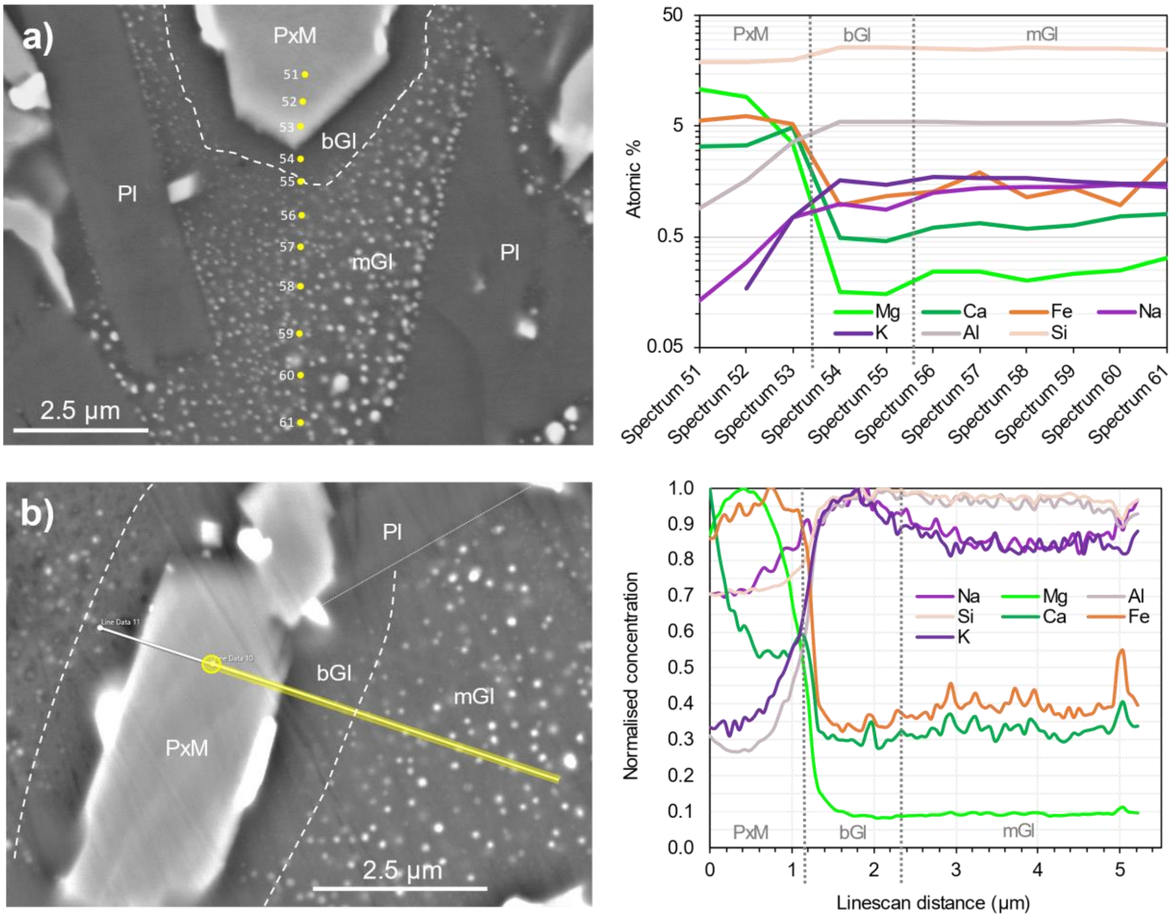

Supplementary Figure 3. **SEM-EDX spot and linescan measurements through the compositional boundary layers surrounding pyroxene microlites.** a) EDX spot measurements starting in the pigeonite and moving linearly through the compositional boundary and into the matrix glass. The elemental abundances for the spectra are plotted to the right of the BSE image with the pyroxene microlite (PxM), boundary-layer glass (bGl) and matrix glass (mGl) boundaries shown as dotted lines. A sharp drop is seen for Mg, Ca and Fe at the boundary layer, before these element concentrations gradually increase in the matrix glass with greater distance from the crystal boundary. Na and K increase in the pigeonite microlite closer to the boundary and are highest in the compositional boundary layer. b) An EDX linescan 5.2 µm long with the point spacing of approximately 80 nm is shown. We plot the high-resolution point spacing against element concentration normalized to the maximum value to better define trends in element concentration across the compositional boundary layer. Within the pigeonite microlite, Al, K, Na and Si increase towards the boundary. Mg sharply decreases, Fe slightly decreases, and Ca slightly increases at the particle boundary. These trends likely reflect diffusion-limited supply of cations from the surrounding glass. Crystal growth continues, but an increasing fraction of less compatible elements (Al, K, Na, Si) are incorporated into the crystal structure to compensate for the lack of available Mg, in particular. In the boundary layer, fewer compatible elements are enriched (due to preferential uptake of more compatible elements). This can be seen well in the relative Na and K concentrations, which are considerably higher in the boundary layer than the matrix glass. Fe, Mg and Ca reach their lowest concentrations in the boundary layer but show only minor increase in concentration with greater distance from the crystal boundary. This may be due to nearby mafic microlites (e.g.., in the bottom left corner) reducing the concentration of compatible elements. All EDX measurements were made using a 10 kV accelerating voltage at a working distance of 10 mm

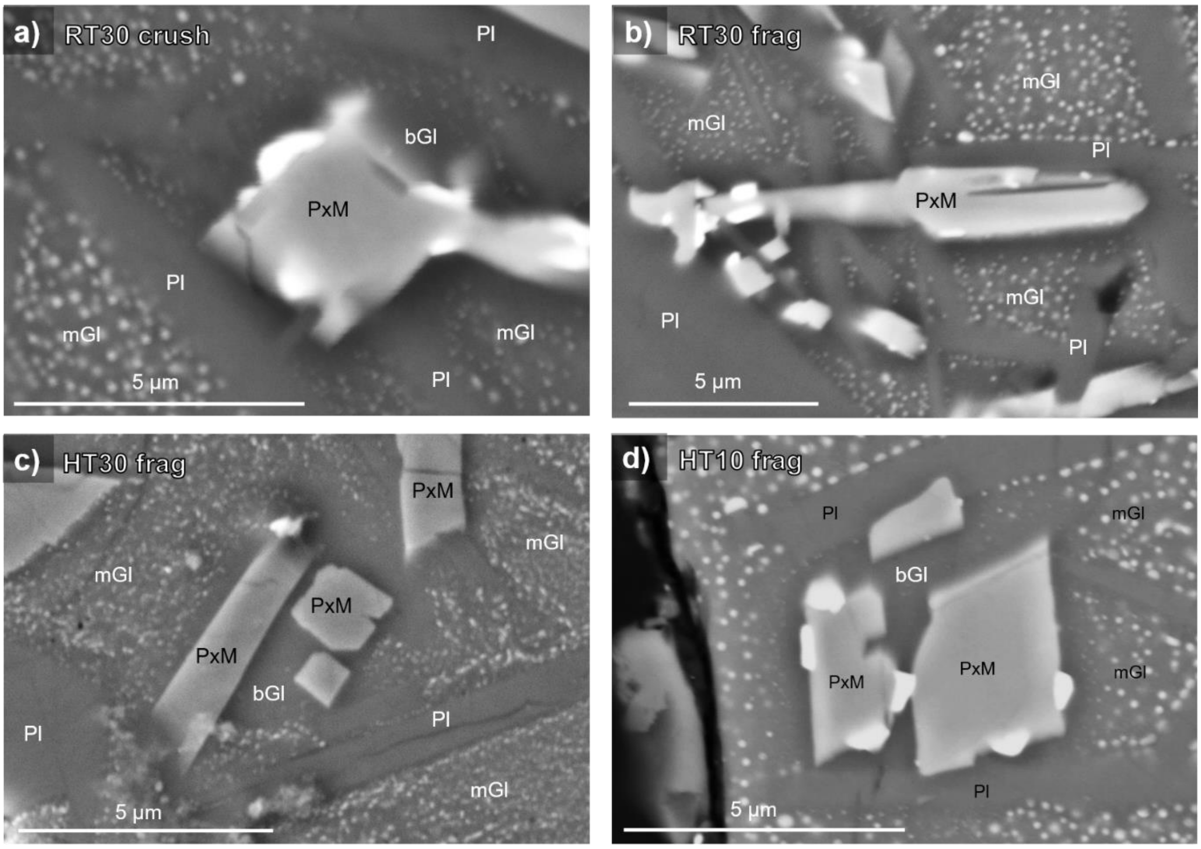

Supplementary Figure 4. **Comparison of textures between pyroclasts formed at room temperature and 850 ºC.** a) and b) show typical microtextures from room temperature (RT) experiments. High-temperature (HT) experiments are shown in c) and d) and exhibit no discernible changes caused by experimental heating. Note that these textures also compare well to the starting material and natural ash particles (Figure 1, Supplementary Figures 5 and 6). Phase abbreviations in the panels follow previous Supplementary Figures.

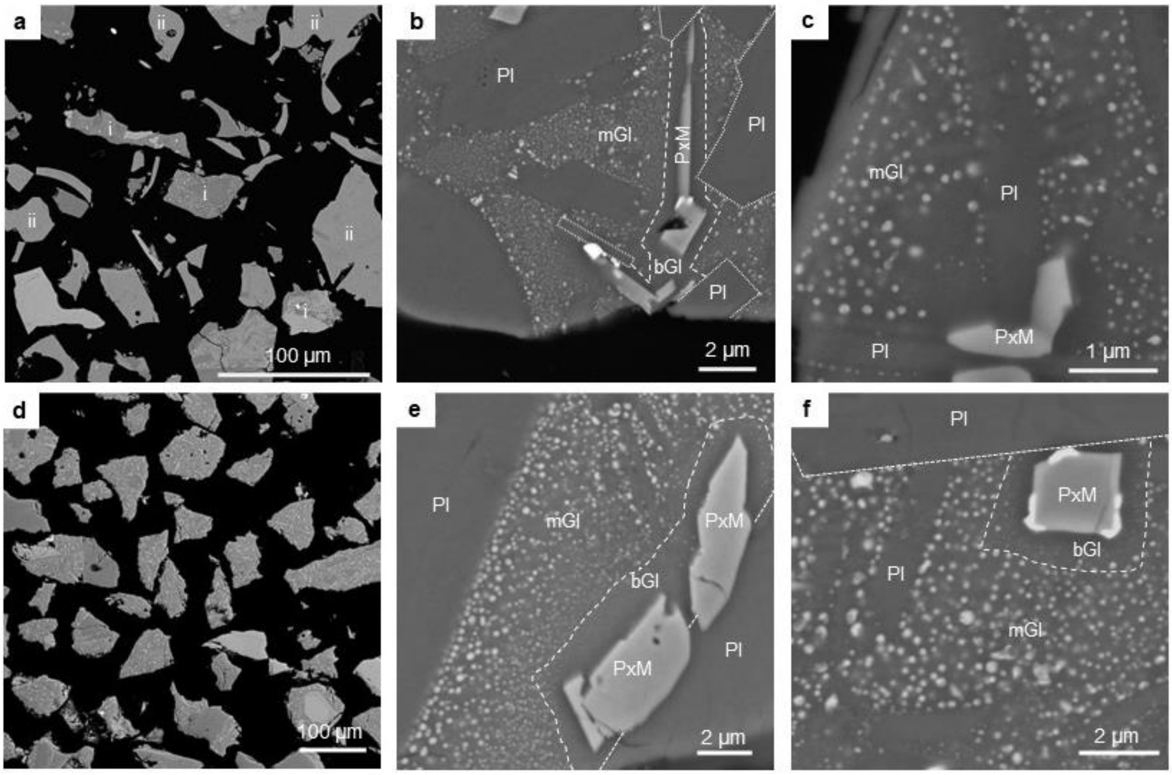

Supplementary Figure 5. A comparison of clast types and textures in natural (a-c) and experimental (d-f) pyroclasts. The two left-hand panels show that natural ash (a) contains grains with similar overall appearance to the experimental texture (d), marked as "i", together with more common smoother-edged grains containing microlite-poor glass with a higher BSE intensity, marked with 'ii'. Microtextures of microlite-rich natural pyroclasts (b-c) are very similar to the experimental pyroclasts (e-f), including bright nanoscale features in the matrix glass (mGl) and single-phase boundary-layer glass (bGl) around pyroxene microlites (PxM). The similar rounded shape and regularly spaced distribution of many of the nanoscale features, together with a gradient in BSE intensity in the bGl (becoming darker toward PxM boundaries), can be seen for both natural (c) and experimental (f) pyroclasts in high-magnification SEM-BSE images.

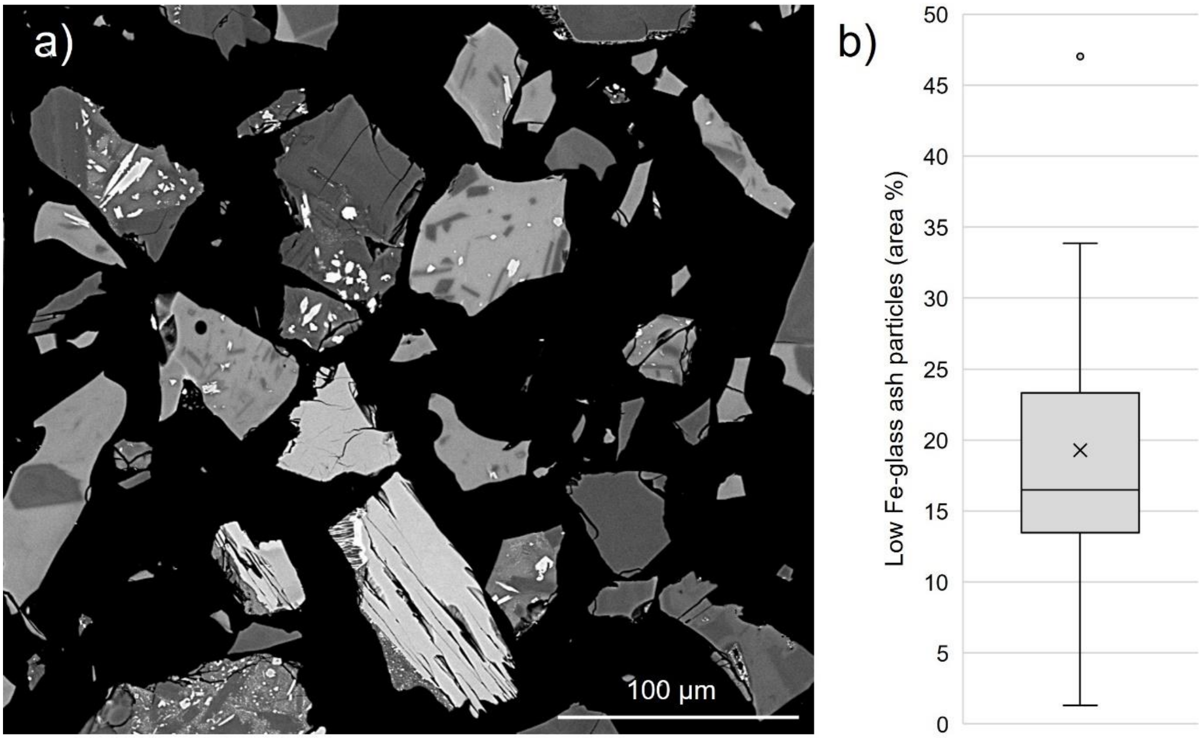

Supplementary Figure 6. Textures of natural pyroclastic fallout samples. a) Cropped SEM-BSE image of particles from pyroclastic fallout deposits of the 16-17th August 2006 eruption. Low-Fe glass bearing particles (e.g. top left particle) have a matrix texture that is indistinguishable from the glass texture in the experimental core samples used in the study. The remaining particles were either matrix-free crystal fragments or relatively high-Fe glass which is attributed to primary magmatic fragmentation leading to the sub-Plinian eruption column formed on the night of 16-17th of August 2006. b) Boxplot representing the area fraction of particles bearing relatively low-Fe, crystal-rich matrix glass in a natural sample from the airfall ash deposits of the 16-17th August 2006 eruption, determined by image analysis of 20 SEM-BSE images of polished stubs at 400x300 µm resolution (with ~30-50 particles per image).

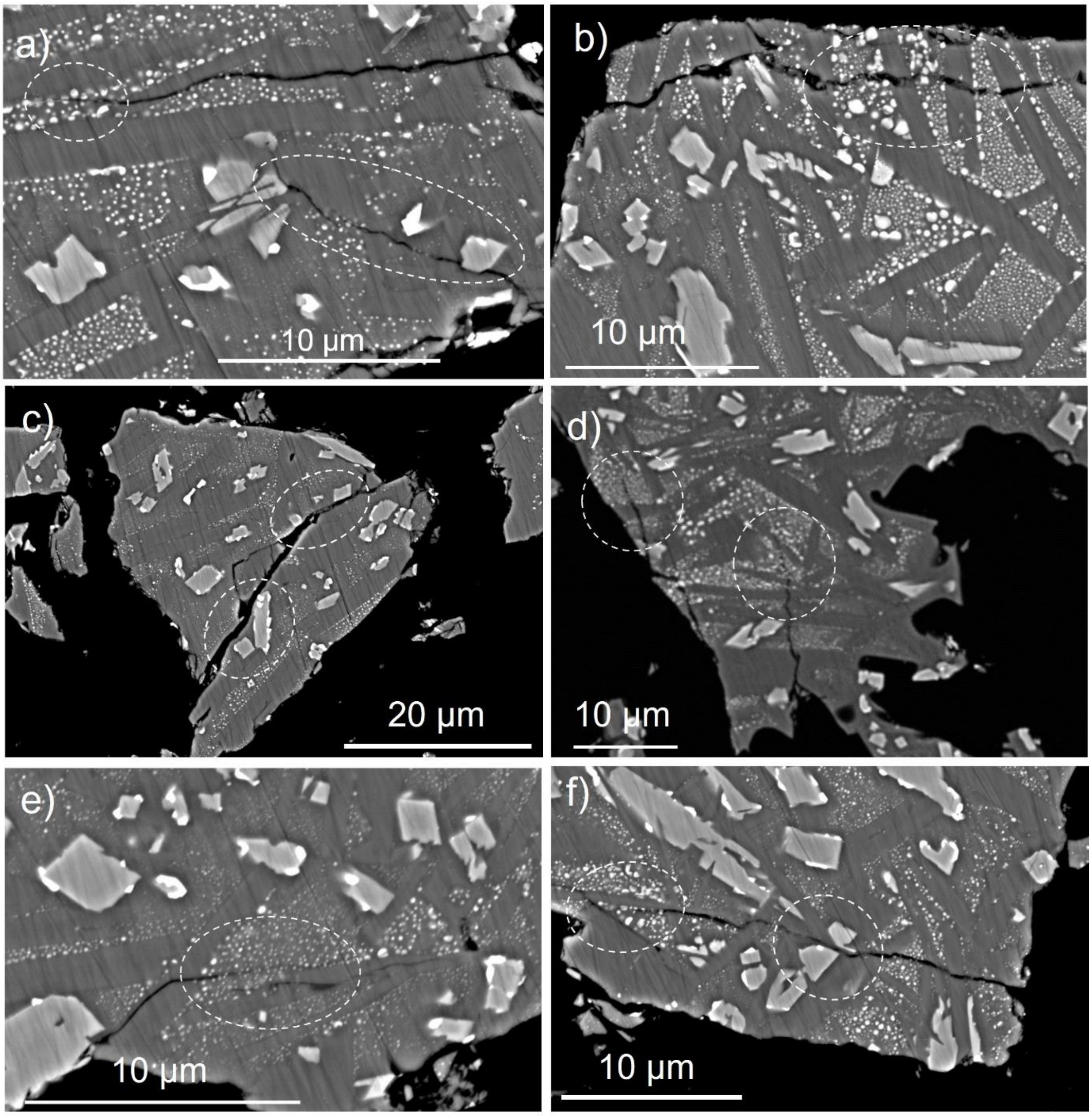

Supplementary Figure 7. Qualitative SEM-BSE observations of fracture pathways in experimental pyroclasts. a) At the bottom-right of the image, a fracture deviates around a pyroxene microlite, then thins twice while passing through small zones of matrix glass, before deviating at the boundary of another pyroxene microlite. b) A fracture appears to deviate around a pyroxene microlite before repeatedly thinning in domains of nanolite rich glass and thickening across plagioclase microlites c) fractures appears to show a preference to pass at, or near, the boundary of pyroxene microlites, rather than through them. Examples are circled. d) Two fractures appear to thin and disappear in droplet-rich matrix (circles). e) A fracture appears to thin and disappear in the matrix glass (circled) before reappearing en echelon along the boundary of a plagioclase microlite. f) Fracture deviation is observed at crystal boundaries, both for pyroxenes (central circle) and plagioclase (further to the left). The fracture also appears to thin and disappear in the matrix glass (circled to the left of the panel), before reappearing along the boundary of a plagioclase microlite.

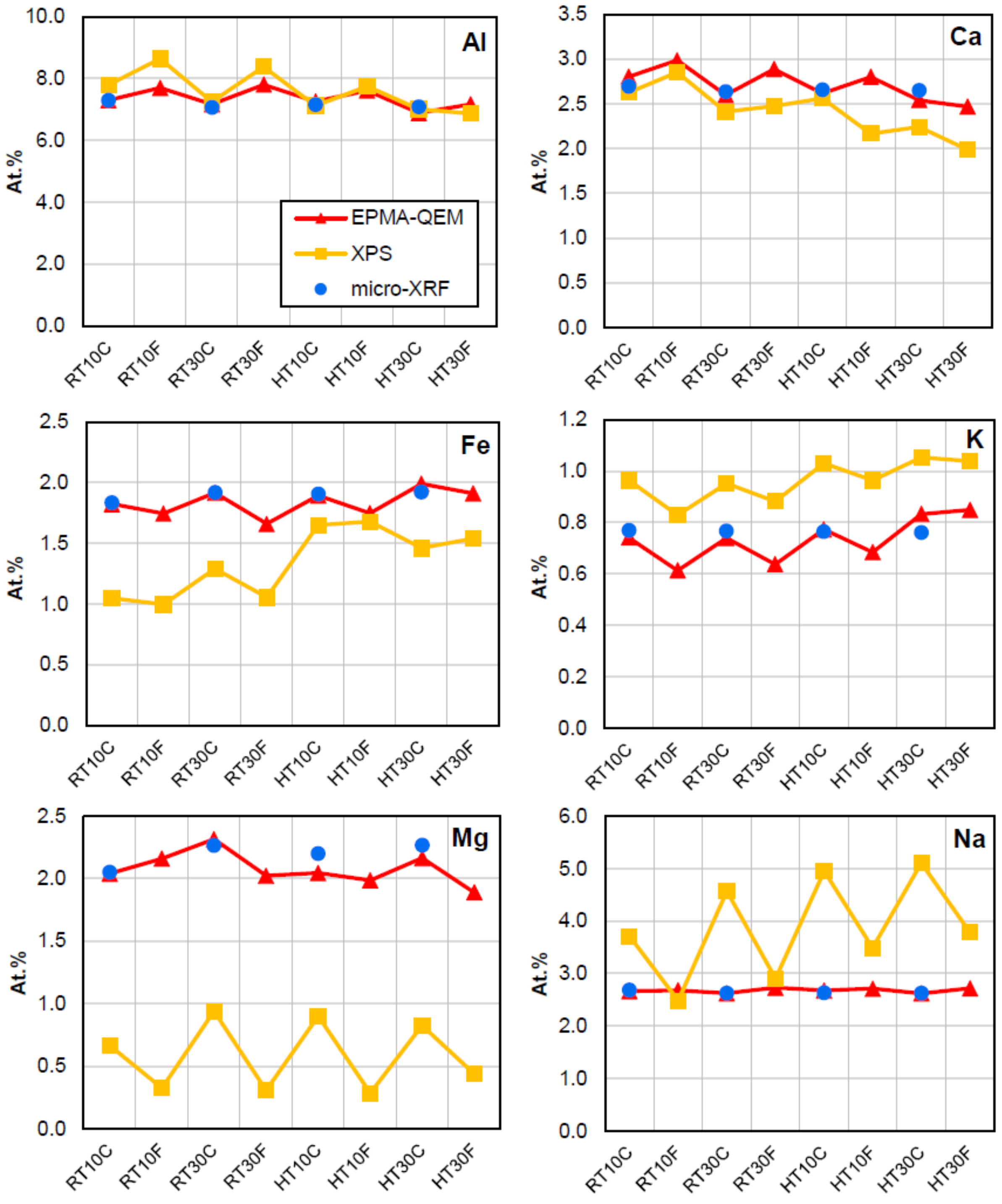

Supplementary Figure 8. Comparison of average elemental concentration from methods used in the study. Average Al, Ca, Fe, K, Mg and Na concentrations in at.% are compared for XPS (yellow squares) and micro-XRF (blue dots) measurements, and calculations based on combined EPMA and QEMSCAN measurements (red triangles) for each experimental sample.

Supplementary Discussion References

1. King, R. P. Mineral liberation. in Modeling and Simulation of Mineral Processing Systems 45–80 (Elsevier, 2001). doi:10.1016/b978-0-08-051184-9.50007-9.
2. Hornby, A. J. et al. Phase partitioning during fragmentation revealed by QEMSCAN Particle Mineralogical Analysis of volcanic ash. Sci. Rep. 9, (2019).
3. Evans, A. G., Dalgleish, B. J., He, M. & Hutchinson, J. W. On crack path selection and the interface fracture energy in bimaterial systems. Acta Metall. 37, 3249–3254 (1989).
4. Mysen, B. O. The structure of silicate melts. Annu. Rev. Earth Planet. Sci. 11 75–97 (1983) doi:10.1146/annurev.ea.11.050183.000451.
5. Andújar, J. et al. Structure of the plumbing system at Tungurahua volcano, Ecuador: Insights from phase equilibrium experiments on july-august 2006 eruption products. J. Petrol. 58, 1249–1278 (2017).
6. Samaniego, P., Le Pennec, J. L., Robin, C. & Hidalgo, S. Petrological analysis of the pre-eruptive magmatic process prior to the 2006 explosive eruptions at Tungurahua volcano (Ecuador). J. Volcanol. Geotherm. Res. 199, 69–84 (2011).
7. Kress, V. C. & Carmichael, I. S. E. Mineralogy and Petrology The compressibility of silicate liquids containing Fe203 and the effect of composition, temperature, oxygen fugacity and pressure on their redox states. Contrib Mineral Petrol vol. 108 (1991).
8. Mysen, B. O. & Virgo, D. Structure and properties of fluorine-bearing aluminosilicate melts: the system Na2O-Al2O3-SiO2-F at 1 atm. Contrib. to Mineral. Petrol. 91, 205–220 (1985).
9. Giordano, D., Russell, J. K. & Dingwell, D. B. Viscosity of magmatic liquids: A model. Earth Planet. Sci. Lett. 271, 123–134 (2008).
10. Wadsworth, F. B. et al. Combined effusive-explosive silicic volcanism straddles the multiphase viscous-to-brittle transition. Nat. Commun. 9, (2018).
11. Weigel, C. et al. Elastic moduli of XAlSiO4 aluminosilicate glasses: Effects of charge-balancing cations. J. Non. Cryst. Solids 447, 267–272 (2016).
12. Tang, L. et al. Effect of nanoscale phase separation on the fracture behavior of glasses: Toward tough, yet transparent glasses. Phys. Rev. Mater. 2, (2018).
13. Jiang, Q. G., Cao, C., Lin, T. C., Wu, S. & Li, X. Strong and Tough Glass with Self-Dispersed Nanoparticles via Solidification. Adv. Mater. 31, (2019).
14. Honour, V. C. et al. Compositional boundary layers trigger liquid unmixing in a basaltic crystal mush. Nat. Commun. 10, (2019).
15. Veksler, I. V., Dorfman, A. M., Danyushevsky, L. V., Jakobsen, J. K. & Dingwell, D. B. Immiscible silicate liquid partition coefficients: Implications for crystal-melt element partitioning and basalt petrogenesis. Contrib. to Mineral. Petrol. 152, 685–702 (2006).
16. Wilke, M. Fe in magma - An overview. Ann. Geophys. 48, 609–617 (2005).
17. Dingwell, D. B. Transport properties of magmas: Diffusion and rheology. Elements 2, 281–286 (2006).
18. Wadsworth, F. B., Witcher, T., Vasseur, J., Dingwell, D. B. & Scheu, B. When Does Magma

Break? in Advances in Volcanology 171–184 (Springer Science and Business Media Deutschland GmbH, 2019). doi:10.1007/11157_2017_23.

19. Cáceres, F. et al. Can nanolites enhance eruption explosivity? Geology 48, 997–1001 (2020).

20. Di Genova, D. et al. In situ observation of nanolite growth in volcanic melt: A driving force for explosive eruptions. Sci. Adv. 6, 413–436 (2020).

21. Di Genova, D., Zandona, A. & Deubener, J. Unravelling the effect of nano-heterogeneity on the viscosity of silicate melts: Implications for glass manufacturing and volcanic eruptions. J. Non. Cryst. Solids 545, (2020).

22. Losq, C. Le, Cicconi, M. R. & Neuville, D. R. Iron in silicate glasses and melts: Implications for volcanological processes. Magma Redox Geochemistry 233–253 (2021) doi:10.1002/9781119473206.ch12.

23. Scarani, A. et al. A chemical threshold controls nanocrystallization and degassing behaviour in basalt magmas. Commun. Earth Environ. 3, (2022).

24. Sparks, S. R. J., Sigurdsson, H. & Wilson, L. Magma mixing: A mechanism for triggering acid explosive eruptions. Nature 267, 315–318 (1977).

25. Pallister, J. S., Hoblitt, R. P. & Reyes, A. G. A basalt trigger for the 1991 eruptions of Pinatubo volcano? Nature 356, 426–428 (1992).

26. Myers, M. L. et al. Replenishment of volatile-rich mafic magma into a degassed chamber drives mixing and eruption of Tungurahua volcano. Bull. Volcanol. 76, 1–17 (2014).

27. Yoshida, K. et al. Oxidation-induced nanolite crystallization triggered the 2021 eruption of Fukutoku-Oka-no-Ba, Japan. Sci. Rep. 13, 1–9 (2023).

28. Clark, A. H. Fe-Ti-P Oxide Melts Generated through Magma Mixing in the Antauta Subvolcanic Center, Peru: Implications for the Origin of Nelsonite and Iron Oxide-Dominated Hydrothermal Deposits. Econ. Geol. 99, 377–395 (2004).

29. Hou, T. et al. Immiscible hydrous Fe-Ca-P melt and the origin of iron oxide-apatite ore deposits. Nat. Commun. 9, 1–8 (2018).

30. Keller, T. et al. Genetic model of the El Laco magnetite-apatite deposits by extrusion of iron-rich melt. Nat. Commun. 13, 1–14 (2022).

31. Cottrell, E. et al. Oxygen Fugacity Across Tectonic Settings. in Redox variables and mechanisms in magmatism and volcanism (eds. Neuville, D. R. & Moretti, R.) 33–61 (AGU Geophysical Monograph, 2021). doi:10.1002/9781119473206.ch3.

32. Cáceres, F. et al. From melt to crystals: The effects of cooling on Fe[sbnd]Ti oxide nanolites crystallisation and melt polymerisation at oxidising conditions. Chem. Geol. 563, (2021).

33. Arzilli, F. et al. Magma fragmentation in highly explosive basaltic eruptions induced by rapid crystallization. Nat. Geosci. 12, 1023–1028 (2019).

34. Matsumoto, K. & Geshi, N. Shallow crystallization of eruptive magma inferred from volcanic ash microtextures: a case study of the 2018 eruption of Shinmoedake volcano, Japan. Bull. Volcanol. 83, (2021).

35. Barone, G. et al. Nanoscale surface modification of Mt. Etna volcanic ashes. Geochim.

Cosmochim. Acta 174, 70–84 (2016).

36. Barone, G., Ciliberto, E., Costagliola, P. & Mazzoleni, P. X-ray photoelectron spectroscopy of Mt. Etna volcanic ashes. Surf. Interface Anal. 46, 847–850 (2014).

37. Ayris, P. & Delmelle, P. Volcanic and atmospheric controls on ash iron solubility: A review. Phys. Chem. Earth 45–46, 103–112 (2012).

38. Cristaldi, D. A., Fortuna, C. G. & Gulino, A. A photoelectron spectroscopy study of lava stones. Anal. Methods 5, 3458–3462 (2013).

39. Delmelle, P., Lambert, M., Dufrêne, Y., Gerin, P. & Óskarsson, N. Gas/aerosol-ash interaction in volcanic plumes: New insights from surface analyses of fine ash particles. Earth Planet. Sci. Lett. 259, 159–170 (2007).

40. Durant, A. J. et al. Long-range volcanic ash transport and fallout during the 2008 eruption of Chaitén volcano, Chile. Phys. Chem. Earth 45–46, 50–64 (2012).

41. Gislason, S. R. et al. Characterization of Eyjafjallajökull volcanic ash particles and a protocol for rapid risk assessment. Proc. Natl. Acad. Sci. U. S. A. 108, 7307–7312 (2011).

42. Berger, G. et al. Experimental exploration of volcanic rocks-atmosphere interaction under Venus surface conditions. Icarus 329, 8–23 (2019).

43. Olsson, J., Stipp, S. L. S., Dalby, K. N. & Gislason, S. R. Rapid release of metal salts and nutrients from the 2011 Grímsvötn, Iceland volcanic ash. Geochim. Cosmochim. Acta 123, 134–149 (2013).

44. Vogel, A. et al. Reference data set of volcanic ash physicochemical and optical properties. J. Geophys. Res. Atmos. 122, 9485–9514 (2017).

45. White, A. F. & Hochella, M. F. Surface chemistry associated with the cooling and subaerial weathering of recent basalt flows. Geochim. Cosmochim. Acta 56, 3711–3721 (1992).
