# Supplementary material for: Nanoscale silicate melt textures determine volcanic ash surface chemistry
Source: Nat Commun. 2024 Jan 15;15:531. doi: 10.1038/s41467-024-44712-6 (PMC10789741; doi:10.1038/s41467-024-44712-6)
Supplement: Supplementary file 3 — Description of Additional Supplementary Files [file 41467_2024_44712_MOESM3_ESM.pdf]

Description of Additional Supplementary Files

Supplementary Data 1 : Electron microprobe measurements of crystal phases within experimental pyroclasts. Pigeonite microlites were found to have two distinct populations, with ~20% number fraction of high-Ca pigeonite (Group A) and ~80% low-Ca and higher Mg pigeonite (Group B). Instrument setup was the same as for glass measurements (see Instrument setup and calibration note below), but with a focused (0 nm) beam.
Total iron was calculated as Fe2+ except in Fe-Ti oxides, where total iron is calculated as Fe3+.

Supplementary Data 2 : Electron microprobe measurements of matrix glass (top) and compositional boundary layer glass (below) from experimental pyroclasts. Due to the defocused beam, it was not possible to measure a single-phase, therefore the measured Fe content is an upper bound. Measurements of boundary glass are smeared, as the defocused beam diameter is greater than the boundary layer width.
All Fe is calculated as Fe^{3+} for glass measurements.
